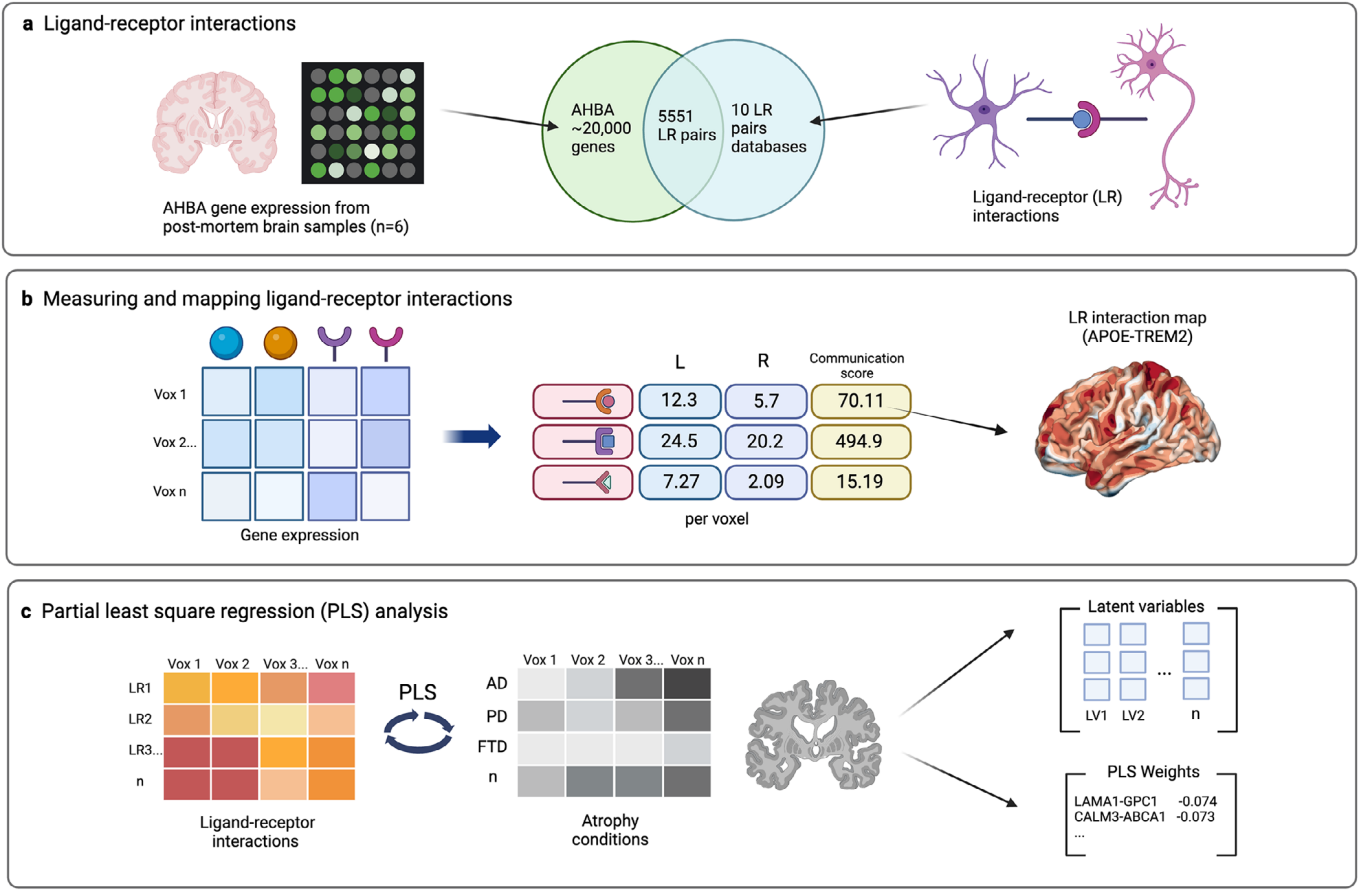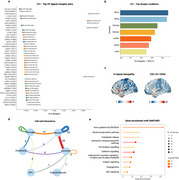# Whole‐brain cell‐cell interaction patterns explain tissue damage in several neurodegenerative conditions

**DOI:** 10.1002/alz70855_099186

**Published:** 2025-12-23

**Authors:** Veronika Pak, Joon Hwan Hong, Gleb Bezgin, Mahsa Dadar, Yashar Zeighami, Yasser Iturria Medina

**Affiliations:** ^1^ McGill University, Montreal, QC, Canada; ^2^ McGill University, Montréal, QC, Canada; ^3^ Montreal Neurological Institute, McGill University, Montréal, QC, Canada

## Abstract

**Background:**

Disrupted interactions among neurons, glial and vascular cell types can lead to inflammation, vascular dysfunction, and neuronal death, highlighting the need to understand how functional interactions between these cells predispose the development of different neurodegenerative conditions. Here we identified cell‐cell interactions across the whole human brain that explain atrophy patterns characteristic to 13 neurodegenerative conditions.

**Method:**

We generated 1,050 whole‐brain neuroimaging maps of ligand‐receptor interactions specific to neurons, astrocytes, microglia, oligodendrocytes, oligodendrocyte precursor cells, and endothelial cells. These maps were created by inferring literature‐curated ligand‐receptor interaction pairs from microarray gene expression derived from post‐mortem tissues of six healthy human donors, sourced from the Allen Human Brain Atlas (Figure 1a‐b). Next, using Partial Least Squares Regression (PLS) analysis, we identified key LR pairs whose patterns of communication explain the spatial distribution of atrophy maps specific to 13 neurodegenerative conditions (Figure 1c). Atrophy maps were previously generated for early‐ and late‐onset Alzheimer's disease (EOAD and LOAD), clinical and pathological subtypes of frontotemporal lobar degeneration (FTLD), Parkinson's disease (PD), dementia with Lewy bodies (DLB), and amyotrophic lateral sclerosis (ALS). Finally, we performed gene enrichment analyses to uncover underlying signaling pathways that explain future atrophy in neurodegeneration.

**Result:**

The first latent variable (LV1) accounted for 84.21% of the covariance (*p* < 0.05), with the COL1A1‐CD36 interaction and other CD36‐associated pairs playing a dominant role in explaining atrophy patterns (Figure 2a). Atrophy patterns common to five FTLD‐related disorders contributed the most to LV1, followed by EOAD and LOAD (Figure 2b). Among the top 10% of ligand‐receptor pairs, 28 of 107 showed strong bi‐directional signaling between astrocytes and neurons, along with prominent contributions from neuron‐microglia and neuron‐neuron signalling (Figure 2d). These top ligands and receptors were significantly enriched for pathways including Slit/Robo‐mediated axon guidance, opioid prodynorphin, enkephalin release, and Alzheimer's disease presenilin pathway (Figure 2e; *p* < 0.001, FDR‐corrected).

**Conclusion:**

We identified whole‐brain ligand‐receptor interactions involved in neuron‐astrocyte, neuron‐microglia, and neuron‐neuron signaling pathways that explain the observed atrophy patterns in multiple neurodegenerative conditions. These key ligands and receptors may serve as potential therapeutic targets and advance our understanding of neurodegeneration.